# Design and Biomimicry: A Review of Interconnections and Creative Potentials

**DOI:** 10.3390/biomimetics8010061

**Published:** 2023-02-02

**Authors:** Alice Araujo Marques de Sá, Dianne Magalhães Viana

**Affiliations:** 1 Department of Design, Institute of Arts, University of Brasília, Brasília 70910-900, Brazil; 2 Department of Mechanical Engineering, Faculty of Technology, University of Brasília, Brasília 70910-900, Brazil

**Keywords:** design, biomimicry, biomimetic design, systematic review, bio-inspiration, bionics, bio-inspired design, nature-inspired design, bio-informed design, sustainability

## Abstract

The study and application of biological knowledge favor the creation of innovative projects in several areas, so it is necessary to better understand the use of these resources specifically in the field of design. Thus, a systematic review was undertaken to identify, describe, and analyze the contributions of biomimicry to design. For this purpose, the integrative systematic review model, called the Theory of Consolidated Meta-Analytical Approach, was used, carrying out a search on the Web of Science with the descriptors “design” and “biomimicry”. For the period from 1991 to 2021, 196 publications were retrieved. The results were organized according to areas of knowledge, countries, journals, institutions, authors, and years. Citation, co-citation, and bibliographic coupling analyses were also performed. The investigation highlighted the following research emphases: the conception of products, buildings, and environments; the exploration of natural structures and systems to create materials and technologies; the use of biomimetic creative tools in product design; and projects focused on saving resources and implementing sustainability. It was noted that there was a tendency for authors to adopt a problem-based approach. It was concluded that the study of biomimicry can stimulate the development of multiple skills in design, improving creativity, and enhancing the potential integration of sustainability into production cycles.

## 1. Introduction

Creative activities, in their most varied manifestations, often adopt interdisciplinary knowledge as a reference for their performance. Thus, from this diversified and integrative approach, the interconnections between the multiple spheres of knowledge are promoted, providing opportunities for innovation. In the opinion of Cardoso [1], the adoption of this type of approach in the processes of teaching, learning, and practice, is essential to expand the range of resources available to designers. It should be noted that, based on the conception of authors such as Bonsiepe [2], Munari [3], and Flusser [4], design consists of envisioning and planning functional creations through the combination of art and technique, giving rise to new forms of culture and expression. Design professionals constantly search for guidelines and methods that enable proposals for products and services that are better adapted to the demands of society. Faced with these enduring challenges, it is essential to explore possibilities and alternatives, such as those that can be offered by biomimicry [5,6].

It is worth mentioning that biomimicry is defined in ISO Standard 18458:2015 as a set of interdisciplinary philosophical and creative approaches that consider nature as a model to guide the development of projects, including aspects related to environmental, social, and economic sustainability. In this field, it is understood that the outcomes implemented by nature provide possible answers to questions of function or practice, simultaneously allowing choices appropriate to the goals of a project. In this way, by observing organisms, biological processes, and ecosystems, solutions can be developed to meet the needs and challenges of the various spheres of human activity, such as in the design of products, environments, services, and digital technologies [6,7,8].

Given the scope of the present work, it is necessary to remark on the persistent impasses regarding the use of terminologies by scholars and professionals involved in creative activities inspired by nature. Some authors adopt the bio-derived terms interchangeably [9]. That is, in a given publication, they globally adopt some bio-derived expressions, which does not allow to sufficiently distinguish the understanding they possessed from the conceptual point of view when structuring and carrying out their studies. Other researchers rely on the specific conceptualization of terms, in line with the ISO 18458:2015 Standard [10,11]. However, for some biomimicry researchers—such as Iouguina et al. [12] and Speck et al. [13]—this field of inspiration in nature has been expanding rapidly, and it is increasingly necessary to differentiate even more clearly and objectively several basic notions, such as biomimicry, biomimetics, bionics, and bioinspiration. For authors who share this concern, even though there are already different understandings of these terms, they are still insufficient. This happens because many authors use different nomenclatures, but, depending on the specific contexts in which each researcher or professional is inserted, these words may reflect cultural factors and aspects of their specializations, which need to be clarified to enhance future work and research. In other words, more theoretical and empirical studies are needed to better delimit the concepts underlying biomimicry. In this sense, review works are very important, despite the advances already achieved. In summary, a close examination of the area reveals that certain biomimetic concepts can be understood as equivalent by certain authors, while, for others, they are perceived as different or complementary [12,13,14]. In view of the above, it is essential to explain that, in the present work, the word “biomimicry” was adopted due to the advantages that still exist when opting for a comprehensive term with wide dissemination. Furthermore, as evidenced by Iouguina et al. [12] and Gamage and Hyde [15], this nomenclature refers to a theoretical and technical framework that can be considered more accessible, in addition to being closely associated with the disciplines of design, architecture, and philosophy.

Additionally, to support the bibliographical research that was undertaken, which will be presented and discussed later, it is worth remembering that a survey of the specialized literature constitutes an essential approach for the systematization of knowledge already gathered, in addition to allowing the identification of trends and to suggesting an agenda for future research, especially in the case of emerging areas of knowledge. Indeed, many review techniques are available (e.g., narrative, integrative, systematic, meta-analyses) [16,17,18].

Among these possibilities is the integrative systematic review model called the Theory of Consolidated Meta-Analytical Approach (TEMAC). This resource, based on bibliometric laws, helps in the development of: research preparation; presentation and interrelation of data; integrative model and validation by evidences. TEMAC is a multilingual model that enables research in various databases, such as the Web of Science, Scopus, and Google Scholar, for reviewing international literature in different areas of knowledge. Furthermore, this resource recommends the adoption of free software such as VOSViewer and TagCrowd for processing and visualization of data. Through TEMAC, it is possible to obtain an overview of the works and productions of an area, to elaborate integrative models, and to carry out comparisons between publications, institutions, and countries [19].

In summary, as the study and application of biological knowledge enables the realization of creative projects in several areas, it is necessary to better understand the use of these resources specifically in design. Thus, in this work, emphasis was given to biomimetic design, also understood as biomimicry-based design, that could be defined as the creative implementation of biologically-inspired concepts, ideas, and strategies into functional products to solve human challenges, that may have the potential to meet the current needs of sustainability in design. This definition is based on the perspectives of authors such as Gamage and Hyde [15]; Hsiao and Chou [20]; Volstad and Boks [21]. Aiming, therefore, to aid the grounding, guiding, planning, creation, and evaluation of biomimetic productions in this field, the general objective of this article was to identify, describe and analyze the contributions of biomimicry to design based on a survey of publications indexed in the Web of Science database.

## 2. Materials and Methods

The investigation was carried out in the Web of Science database, using the descriptors biomimicry and design. The period between 1991 and 2021 was configured as the time range for the searches, totaling three decades. It should be noted that the first decade of this interval coincides with the publication of the work by Benyus, Biomimicry: Innovation Inspired by Nature, which contributed to the dissemination of the term “biomimicry” [22]. To carry out the systematic review, the Theory of Consolidated Meta-Analytical Approach (TEMAC) integrative model was adopted. This resource recommends organizing the information collected in the database according to areas of knowledge, countries, journals, institutions, authors, and years. In addition, TEMAC guides the performance of citation, co-citation, coupling, co-occurrence, and word cloud analyses with the generation of images, such as heat maps and network or line maps. For this purpose, the VOSViewer and TagCrowd platforms were used [23].

It is worth clarifying that citation analysis is one of the means of measuring the impact of authors’ production based on the number of citations listed in the database. Evidently, it is important not to mistake the popularity of an author or publication with the relevance of the scientific work developed, but it is an interesting comparative parameter. The co-citation analysis corresponds to works that are frequently cited together by other publications. The greater the number of co-citations of a document, the greater its prominence in the literature, and the more likely its semantic relationship with the other two documents will be. Coupling establishes that when two documents cite the same third publication in their bibliographies, this is an indicator that these works have similarities [19,23]. 

In TEMAC, it is common to delimit longer periods for co-citation than for coupling, since the first type of analysis is adopted to highlight the most-used thematic approaches in the study of a given topic (comprehensive panorama), while the second type, focuses on reduced intervals and, therefore, tends to show the main fronts of contemporary research [19,23,24]. Therefore, in the present work, co-citation covered the period from 1991 to 2021, and coupling comprised the years from 2018 to 2021. Bearing in mind these methodological guidelines, interrelationships were established between the data obtained with the help of TEMAC and the content of the identified works.

## 3. Results

Preliminarily, 633 documents were obtained. It is considered essential to point out that, as biomimicry is a recent area—especially when associated with the field of design—various types of documents were examined in the present literature review, including articles, publications in proceedings of events, and books. The research of such works is pertinent because they represent important sources of information and can contain and record discoveries in emerging areas.

The oldest work found on the Web of Science was the article by Li et al. (1995), in which the authors explored structural applications of synthetic composites and natural bamboo fibers [25]. The material produced showed mechanical behavior similar to resistant structures present in nature. Considering this pioneering study with a biomimetic approach, it is pertinent to highlight that the initial works on the subject were directed mainly at the study of materials from an engineering perspective (especially at microscopic and nanoscopic scales), including physicochemical and structural investigations. With regard to pioneering efforts and research, it should be noted that studies and applications, more clearly related to the areas of design and architecture, followed in greater numbers after the publication of the book by Janine Benyus (1997), that is, from the end of the 1990s [22].

Based on the examination of previous evidence, a filter according to knowledge areas was implemented in the subsequent step of the review. Thus, divergent categories were excluded (e.g., agriculture, medicine, dentistry, physics, chemistry, biochemistry, materials science, electrical engineering, computer science, mathematics, nanoscience, nanotechnology, pharmacy, and neurosciences). This reduced the number of selected works to *n* = 196. Table 1 presents the analysis categories used to process the information and the respective quantitative data obtained from the search on the Web of Science. In this set of documents, it was noted that the areas of knowledge with the highest number of contributions, in descending order, were: engineering (*n* = 64), the science of technology (*n* = 41), environmental sciences, and ecology (*n* = 31), and architecture (*n* = 24). It is vital to mention that the Web of Science does not have a specific category for filtering documents published exclusively in the domain of design, unlike other areas of knowledge. This limitation impacts the investigation and means that works of interest in this field are obtained only in related areas. 

The three countries with the highest number of publications were, respectively: United States (*n* = 68), United Kingdom (*n* = 19), and Turkey (*n* = 15). The following journals published the highest number of works on the subject: WIT Transactions on Ecology and the Environment (*n* = 9), Scientific Reports (*n* = 6), Architectural Science Review (*n* = 5), and Procedia Social and Behavioral Sciences (*n* = 5). Regarding the institutions associated with publications about interconnections between design and biomimicry, the following stood out: University of California System (*n* = 8), Victoria University of Wellington (*n* = 8), University System of Georgia (*n* = 7), Delft University of Technology (*n* = 6), University of Akron (*n* = 6). It was also found that Maibritt Pedersen Zari was the author with the most published works (*n* = 8), followed by Donnison, Faludi, Jones, Niewiarowski, and Pauw (*n* = 3). The years with the greatest profusion of works were: 2019 (*n* = 27), 2016 (*n* = 26), and 2020 (*n* = 24).

In order to provide an overview of the bibliographic production regarding design and biomimicry analyzed in the review reported here, Table 2 presents a summary of publications that showed 35 or more citations in the Web of Science between 1991 and 2021. 

Examining the table, it is possible to observe that the article “Biomimetics: lessons from nature—an overview” [26] was significantly more cited (*n* = 691) than “Biomimicry in textiles: past, present, and potential—an overview” [27], the work with the second highest number of citations (*n* = 81). It should be noted that the work of Bhushan (2009) highlighted the field of biomimetic research at a microscopic and nanoscopic scale to generate creations inspired by biological surfaces and materials. The author presented sets of inspiring natural elements addressing possible applications in products, considering aspects of: superhydrophobicity, self-cleaning, fluid flow, drag reduction, adhesion, aerodynamics, structural coloration, thermal insulation, and mechanical strength.

**Figure 1:**
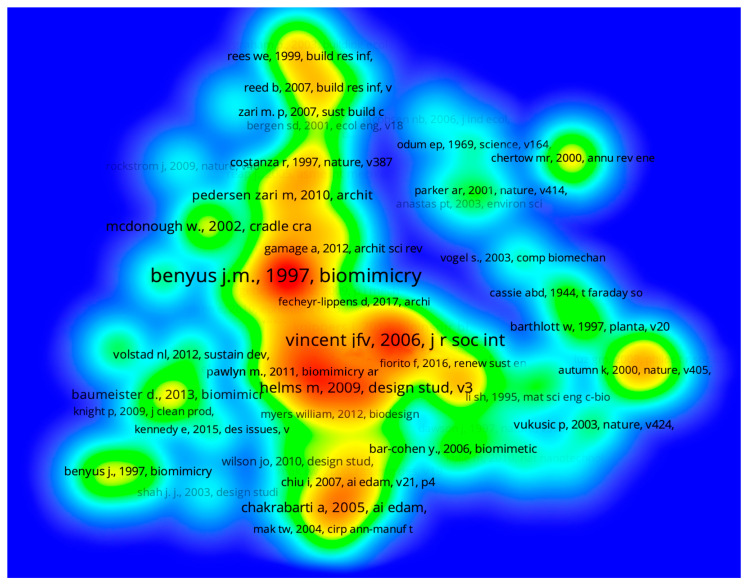
Co-citation map generated from data obtained in the Web of Science, covering the period from 1991 to 2021. Source: the authors.

To identify the main thematic emphases adopted in Biomimicry research and projects oriented toward design, a heat map of co-citations was prepared. This analysis determines which documents are frequently cited together to identify the highest-density nuclei that highlight authors and works with similar lines of research. Thus, it is possible to map the proximity of studies and ascertain their thematic and theoretical approaches [19]. In Figure 1, the larger the typeface size of the names and the redder the areas where they are inscribed, the greater the concentration of each nucleus.

The layout of the co-citation map reveals that the cluster encompassing Benyus (1997) shows the greatest convergence [22]. This was an expected result since this work constitutes one of the fundamental publications on biomimicry and contributed to consolidating the subject as a field of research. As is widely known, in her work, Benyus conceptualized nature as the “model, measure and mentor” agent, capable of generating complex systems of high efficiency and economy, encouraging the discovery of solutions based on the transfer of natural knowledge to the human context, especially for the challenges related to material disposal, circulation of energy, resource-saving and problem management.

In Figure 1, another significant nucleus revolves around Vincent et al. [9]. In the aforementioned article, the authors emphasized that biomimicry requires specific tools and processes. One possibility highlighted by Vincent et al. is the use of the TRIZ heuristic matrix, which can be adapted for biologically-inspired projects in the BioTRIZ tool. In it, notions obtained from natural systems are used, transferring configurations to conceive innovations in the creation of technological systems and projects, mainly with the inclusion of energetic and structural aspects based on nature.

The publication by Helms, Vattam, and Goel [35] about the naturally inspired approaches that integrate analogy systems to develop solutions in product design lies in this same cluster. This study featured the two main approaches adopted in biomimetic projects: one centered on the design problem (known as design for biology or problem-based approach) and another, which starts directly from the study of a natural aspect (known as biology to design or solution-driven approach). This article also cautioned against common mistakes in biomimetic design practices, that should be avoided throughout the creation process, such as: a vague and overly broad definition of design problems; the inappropriate combination of design proposal and selection of the biological element; the use of superficial natural analogies; the inappropriate simplification of complex biological functions; the use of pre-formulated solutions without examining the project context; and preference for the first solution found, without analyzing alternatives, either for biological inspiration or for detailed design proposals.

Next to this group of works is the publication by Pawlyn [36], which showcased the interconnections between biomimicry and architecture, seeking technological projects of sustainable and innovative construction that may also have a restorative character for environments, based on conscious and systemic initiatives. Supported by various examples and cases, the author emphasized the importance of considering: structural efficiency; mindful use of resources; understanding manufacturing processes; analysis and implementation of zero-waste systems; clean energy generation; and water saving.

In the lower central portion of the co-citation map are Chakrabarti et al. [37], whose article presented the GEMS of SAPPhIRE Model of Causality tool, which contributes to the functional comprehension and definition in biomimetic design projects. This model enabled the development of a computational tool and a database that help designers find solutions for their projects based on analogies of natural and artificial systems (IDEA-INSPIRE). The authors concluded that natural systems have been insufficiently adopted as a source of inspiration for design projects, unlike artificial systems. Thus, their proposal is very relevant to the field of biomimetic product design and the systematization of idea generation.

Other publications, shown in Figure 1, are also worth mentioning. Located in the lower left part of the co-citation map, is the Biomimicry Resource Handbook manual by Baumeister et al. [38], which provides the essentials of biomimicry, including guidelines for applying biomimetic methods, approaches, and tools to conduct creative projects (e.g., Biomimicry Thinking Design Lens, Challenge to Biology and Biology to Design approaches, Biomimicry Taxonomy Chart, Life’s Principles Diagram).

Another interesting work found on the map is Cradle to Cradle by Braungart and McDonough [39], which is positioned in the upper left portion of the map. This publication proposes reinventing the life cycle of products and processes. In their work, the authors emphasized the integration of design and science to favor lasting social benefits, stimulating the regeneration of the natural realm and the improvement of human quality of life. They also advocated the use of safe materials, water, and energy in the context of the circular economy, eliminating the concepts of waste and disposal. Therefore, according to Braungart and McDonough, design can be reconfigured with a view to effectively having a positive impact.

In view of what was previously presented, through the analysis of the co-citation map, it became possible to verify the thematic emphases of publications in the period between 1991 and 2021. As expected, the main nucleus was concentrated around the work of Benyus, which was a crucial work in the dissemination of knowledge about biomimicry. In addition, it was found that, the central area of the map contained nuclei related to the development of biomimetic design works in which the authors present field theories and project cases that exemplify the use of biomimicry principles. It is interesting to mention that, on the map, there was also a highlight on clusters whose publications evidenced specifications of technical aspects, including development stages and processes of biomimetic design. This was carried out through the presentation of tools, models, approaches or processes, and guidelines for projects. Some of the works of this group are directed toward the research and functional analysis of natural and artificial systems. Finally, smaller nuclei, whose works discuss issues of circularity and sustainability, which are often related to biomimicry-based design, are visible.

It is worth mentioning that a predominance of the problem-based biomimetic approach was found in the works of multiple authors located in the center of the map. Such an approach begins with a design challenge and the functional requirements of a project, and only in the following steps of the creative and research processes are investigated biological aspects relevant to each context. As previously stated, some of these authors have also proposed and made available processes and tools to stimulate biomimetic design projects that are primarily problem-based.

**Figure 2:**
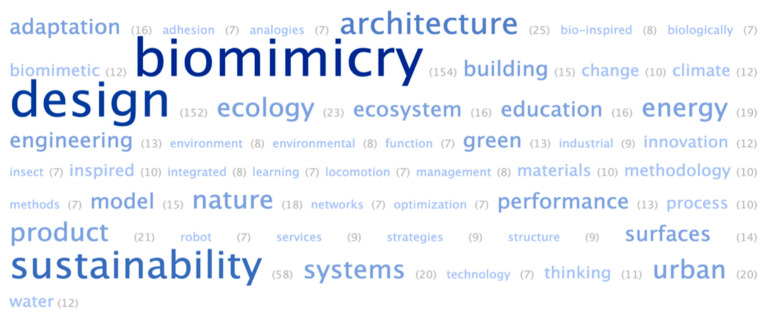
Word cloud generated from data obtained in the Web of Science, covering the period from 1991 to 2021. Source: the authors.

After performing the co-citation analysis, a word cloud was generated (Figure 2) composed of the fifty most frequent words from the set of works published in the period from 1991 to 2021 (*n* = 196).

In this illustration, it can be seen that, in addition to the descriptors used in the present research, the most frequently mentioned keywords were, respectively: “sustainability” (*n* = 58), “architecture” (*n* = 25), “ecology” (*n* = 23), “product” (*n* = 21), “systems” (*n* = 20), and “urban” (*n* = 20). Thus, sustainability would seem to be one of the main research themes in design and biomimicry, whether for the development of products, buildings, or cities. In addition to these words, it is essential to reflect on the entire image, which includes terms such as “adaptation”, “systems”, “networks”, “ecology”, “environment”, “climate”, and “change”, which are associated with the adaptive approach at a systemic level, that in turn can reveal important technical solutions, especially in the face of the climate crisis, contributing to the generation of connected, adapted, and resilient projects. The words “education”, “methodology”, “methods”, “performance”, “product”, and “optimization” are also included, and denote concerns about the efficiency and functional improvements in the creation of biomimetic products, including resources associated with its practice and teaching. 

Examining the image again, it is possible to identify words that, at first glance, appear to be distant from the other groups of terms, such as: “insect”, “water”, “locomotion”, and “adhesion”. Such results probably come from research that emphasizes the morphology and movement of organisms, especially in the field of entomology. The water environment is also an interesting result as it can be associated both with new hydrodynamic, hydrophilic, or hydrophobic products, such as surfaces and vehicles, for example; and also, with products for water harvesting, or with the study of aquatic organisms and their characteristics. The term “adhesion” represents another specific focus of research in biomimicry, directed toward the creation of new materials and surfaces with bioinspired properties at the microscopic and nanoscopic levels.

**Figure 3:**
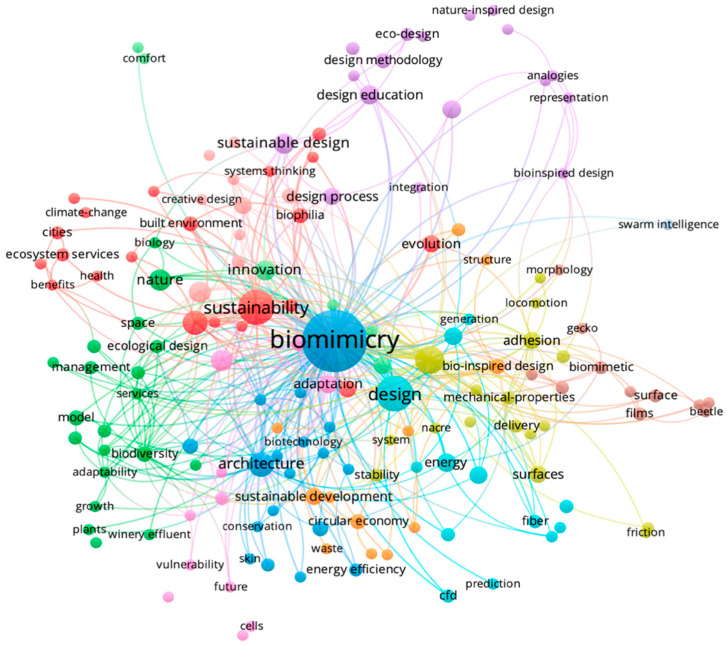
Co-occurrence map generated from data from the Web of Science platform, covering the period from 1991 to 2021. Inspecting the colored sets, it is possible to identify a total of nine different groups. Source: the authors.

The next step was the co-occurrence analysis, carried out with the words of the group of publications corresponding to the period from 1991 to 2021 (*n* = 196). The VOSViewer software, used to generate Figure 3, automatically assigned different colors to distinguish each thematic grouping of words. The interconnections between words and groups are established through the interweaving of colored lines. The closer to the center of the diagram and the larger the typeface size, the stronger the connections between groups and terms and the greater the prominence in the researched literature.

It was found that, similarly to what was exposed by the word cloud analysis, the terms “sustainability”, “sustainable design”, and “architecture” are in evidence at the center of the thematic clusters, in proximity to the descriptors of this research. The red grouping emphasizes sustainability; promotion of well-being and health associated with nature-inspired initiatives and biophilic configurations. It also includes issues related to climate change and ecosystem services. In the green group, there are words about building models and innovative proposals based on natural aspects and ecological design, including biodiversity concerns. In both blue groups, the areas of design and architecture are evidenced, as well as the exploration of biotechnologies and the study of energy efficiency and materials such as fibers. The pink set illustrates a concern for the future and the need to integrate adaptive criteria into projects. In orange, words focused on principles and practices of sustainable development, waste management, and circular economy stand out. The brown cluster refers to the morphological study of animals such as geckos and beetles. The group in yellow is directed toward investigations on materials and surfaces (e.g., adhesion properties, locomotion). In the purple group, themes linked to the exploration of design methodologies and processes interconnected with sustainability factors, eco-design, bio-inspired design, and the use of creative analogies can be found.

It is interesting to note that terms related to sustainability were distributed in more than one colored group. It can be inferred that this topic is one of the notable potentials of biomimicry creations and technologies. As such, it permeates many different subjects related to research and production of biomimetic design including a diversity of application scales (city level, construction, object, materials, and surface properties). 

**Figure 4:**
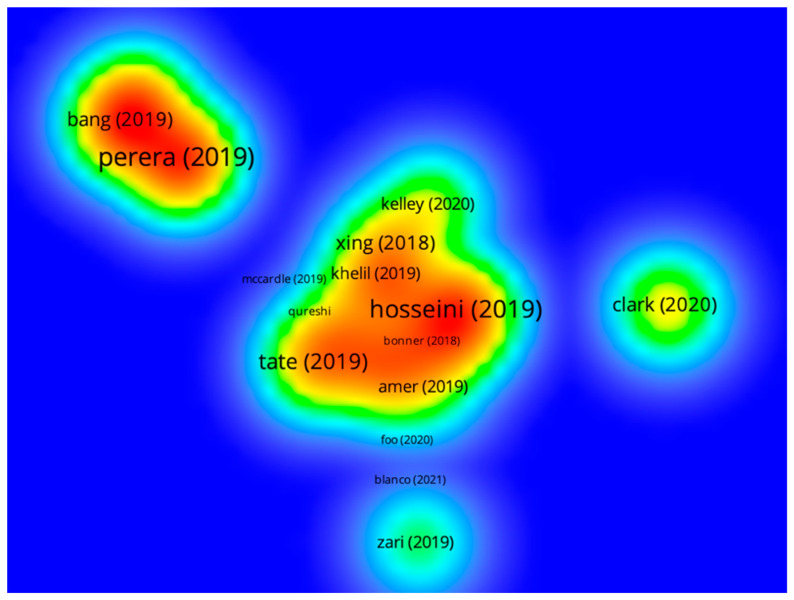
Bibliographic coupling map created from data obtained in the Web of Science, covering the period from 2018 to 2021. Source: the authors.

Once the citation, co-citation, co-occurrence, and word cloud prospecting stages were completed—all referring to the period from 1991 to 2021—another temporal filter was used to carry out the bibliographic coupling analysis (years from 2018 to 2021), obtaining a total of 78 publications. It is important to recall that TEMAC recommends the performance of coupling analysis to investigate recent research fronts [19,23]. Below, Figure 4 shows a representative heat map of this assessment, in which research emphases can be observed during the defined period.

On the left of the coupling map, in a high-concentration nucleus, is the publication by Perera and Coppens [40]. The authors investigated new design possibilities through the lens of bioinspired materials and chemical engineering. Some of the presented materials were based on the structure of mother-of-pearl; on the strong, reusable adhesives that mimic gecko toe-pads; and on the antibacterial surfaces based on shark skin. Perera and Coppens emphasized that seeking inspiration in natural patterns and microstructures provides an opportunity for redesigning materials, processes, and products which may result in creations that, even without directly resembling the mimicked organism, will have an inherent link with nature in their properties and functionality.

The red-colored area, in the center of the map, groups the works by Hosseini et al. [41], Tate et al. [42], Xing et al. [43], and Amer [41]. Hosseini et al. [41] gathered literature on kinetic and adaptive architectural façades that act as permeable complex interfaces between indoor and outdoor environments. The authors analyzed the ability of these elements to function as a protective layer responsive to climatic variations. They proposed a design process to guide the construction of kinetic façades from a morphological and interdisciplinary approach, in which biomimetic characteristics are combined with parametric and energy efficiency technologies.

Tate et al. [42] studied the interaction between biomimicry and the circular economy based on ecosystem analogies. They called attention to the flows of energy and matter in nature, primarily based on recycling and reuse between the different trophic levels. Tate et al. exemplified their concepts by reviewing relationships of mutualism found in nature. In the authors’ opinion, the insertion of biomimetic knowledge and principles into new economic and business approaches can promote innovation in the organizational sphere. In fact, this work contributed to the recognition of biomimicry as an area from which knowledge can be useful in the strategic, administrative, and organizational plans of companies.

Xing et al. [43] combined the study of plants, more specifically plant cells, with the development of façade design and technologies for building cladding, resulting in a conceptual proposal for a biodome, with improvements in thermal insulation and energy efficiency. The authors prioritized the study of plant-based analogies to generate constructive proposals, due to the sessile character of plants, which requires them to adapt to the surrounding conditions (as they fixed in a single location, a characteristic also associated with buildings). Xing et al. generated a framework for the design process of biomimetic projects.

Amer [44] incorporated biomimetic notions into the syllabus of a university course, covering the following topics: interrelationships between biomimicry and building design; project methodology; natural lighting management; and plant-inspired façades; as well as research activities, design, parametric digital prototyping (with Rhinoceros and Grasshopper software), and the use of the biomimetic design concept generation matrix. According to the author, the students realized the importance of the responsibility inherent in professional practice regarding the preservation of the health of human communities and those of other species.

Additionally, in Figure 4, two more nuclei can be observed, with the works of Clark [45] and Zari [46]. The first publication researched marine biomineralization for monitoring and forecasting the impacts of climate change and for the design of new biocomposite materials [45]. In the second publication, Zari [46] endeavored to analyze how the urban environment can insert new proposals into its design that incorporate ecosystem services committed to the regeneration of biodiversity and the resilience of natural environments faced with climate change.

In summary, an examination of the results from the coupling analysis allowed verification that the themes of recent research in biomimicry and design include, on one hand, the investigation of the physicochemical properties of materials and microstructures, mainly biological structures of reptiles and marine animals. On the other hand, there is a focus on the design of components, coatings, and finishes for application in architecture, including bioclimatic factors (e.g., local context, energy, and resource savings) and environmental responsiveness. This enables envisaging the creation of adaptable buildings that operate similarly to living organisms. In this scope, the authors mainly investigated plant elements as a source of inspiration. Among the highlighted works, there were also many proposals for creative tools, frameworks, and parametric software that aim to assist in the search for biological solutions and the transfer of such natural characteristics to the conceptual development in design. Another research emphasis focused on sustainability, including aspects of ecosystems and interspecific ecological relationships that demonstrate the potential to inspire a multiplicity of projects, such as the design of products and even strategic design. In addition, in the present study, there were research perspectives that addressed, respectively, the importance of supporting ecosystem services in the urban environment and the development of materials such as biocomposites. 

Given what is presented above, it is possible to infer that the coupling analysis highlighted initiatives in biomimetic design that demonstrate a remarkable capacity to generate innovations to solve emerging challenges in several fields of human activity. Finally, it should be stressed that, once again, the authors highlighted in the densest cluster produced research and projects guided by the problem-based approach.

## 4. Discussion

The results of this systematic review allowed establishing connections and making conjectures about the scenarios of publications indexed by the Web of Science. It is believed that the panorama of productions showcased in the present research encompasses contributions that may assist researchers and designers in their endeavors, as the main themes and emphases of projects and research were identified in the investigated literature, representing possible paths for new creations and studies.

The analysis of the data obtained through this survey indicated that the works involving design and biomimicry are associated with the following thematic aspects: (a) the conception of products, buildings, and environments; (b) the exploration of structural and systemic conformations of nature for the production of new materials and technologies; (c) the use of biomimetic creative tools to guide idea generation in product design; and (d) biomimetic creations with an emphasis on resource savings and sustainability.

It was also noted that innovations which correlate design and biomimicry have been applied in different domains, namely: health (e.g., new equipment); textiles (e.g., fibers and fabrics); robotics (e.g., programming and navigation); materials science (e.g., adhesives and self-cleaning materials); construction (e.g., kinetic façades and structures); and environmental science and ecology (e.g., ecosystem services, regeneration of biodiversity). In this sense, it is pertinent to mention that the publications, which were listed and organized in this review, reflect development in several areas of cutting-edge technology, which is consistent with the innovative tendency intrinsic to the field of biomimicry.

However, it is relevant to underscore that there is still a scarcity of works oriented toward the development and use of everyday objects and in the field of services, given that these are two areas of great importance in the design domain. In view of this finding, it is reasonable to assume that more efforts and investments are required in design projects that clearly and consistently recognize their foundation in the principles of biomimicry. Thus, it is crucial to more frequently adopt a biomimicry perspective to research and develop products intended for the daily lives of consumers, in order to guarantee a better quality of life for the users of these objects (e.g., bio-inspired functional and technological improvements of products). This limitation is even more alarming upon verifying that most of the publications retrieved from the Web of Science originate from engineering and architecture schools and departments. As a result, there are not enough projects produced by teams from schools and departments associated exclusively with the field of design. It is possible to assume that the absence of the ‘design’ filter when searching the Web of Science may constitute a restriction for specific dissemination of works.

Another trend observed in the present review, highlighted by the analysis of the heat maps, concerns which type of biomimicry-based approach the authors adopted in their projects and research. The results indicated that many of them started by addressing the problem and the required function (problem-based approach) instead of beginning directly from the investigation of natural aspects (solution-driven approach). It should be clarified that this could be expected since the survey encompassed the field of design, an area where projects and creative processes commonly begin with the definition of a problem or a challenge associated with human activity. However, supporting the development of more projects and research in design based on the solution-driven approach can also be of great interest, as it can contribute to enhancing and refining creative skills. In addition, this approach contributes to establishing connections with specialists from other areas (e.g., biologists) and may also help bring designers closer to field research in nature.

Regarding design processes, efforts were made to provide information about biomimetic tools that assist in the planning and execution of creative proposals (e.g., Biomimicry Thinking Design Lens, Biomimicry Taxonomy Chart, BioTRIZ, GEMS of SAPPhIRE, IDEA-INSPIRE, BECE-Framework) [9,15,30,35,37,38]. Such instruments, connected to the problem-based approach, mainly focus on the conceptual development phase of the project, supporting the investigation of characteristics of nature in their transposition into the design domain. In addition, various digital resources were explored by the authors to generate complex shapes and patterns for models and products (e.g., parametric software and rapid prototyping). The use of biomimetic tools in creative design processes, reported in the analyzed literature, can still be considered restricted, given that there was not enough detail on the application of such instruments. Consequently, it was not possible to verify, in this review, whether the authors resorted to other resources or documents, or if they deliberately chose not to include this information in the communication of their works. 

The TEMAC model contributed to the identification of the main themes, approaches, and trends in the literature. Through it, it was possible to infer which areas are expanding and which deserve to be further investigated. The tool also allowed comparisons to be made between the works and production of different authors, countries, and institutions. In addition, the recommendation of TEMAC to adopt software, such as VOSViewer and TagCrowd, enabled the graphical representation of the results (e.g., heat maps and line maps), which facilitated carrying out the analyses, in addition to illustrating them satisfactorily. However, like any procedure for surveying and analyzing the literature, it is crucial to understand that, from the moment specifications are established for the research themes, descriptors, software, databases, and filters that will be used, it is inevitable that certain works will not be captured by the selected criteria, which can limit the scope of the contributions of a review such as the present work.

The chosen database for this research (Web of Science) is one of the main multidisciplinary repositories, having an excellent temporal coverage of publications, in addition to ensuring a frequent update of indexed works, including complementary materials such as conference proceedings [19]. Nevertheless, the Web of Science mainly covers content published in English and, as discussed earlier, there is no specific filtering function for the field of design. Thus, the investigation of other multilingual repositories, such as Scopus, Google Scholar, EBSCO, ProQuest [19,47] is recommended. Furthermore, when analyzing other systematic reviews, it is noticeable that other software and platforms, in addition to those employed in the present review, have been used by authors to organize and facilitate data processing. Some examples are: MAXQDA, CiteSpace, Publish or Perish, San-keyMATIc, Bibexcel, Gephi, Pajek, Ucinet, and Science of Science (Sci2) [47,48,49].

The adoption of the descriptors biomimicry and design was sufficient to achieve the objectives proposed in the scope of this research. However, considering that only two descriptors were used, it is suggested that more terms be incorporated in future surveys, such as: bioinspiration, biomimetics, bionics, biodesign, biomimetic design, bioinspired design, bionic design, nature-based design, nature-inspired design, biologically-inspired design, and bio-informed design. Thus, it will be possible to cover an even greater number of works, which may lead to relevant developments for the scientific and professional communities.

In summary, with this review, it is necessary to emphasize the importance of adopting interdisciplinary knowledge in the field of design to expand the creative perspectives of professionals and to diversify theories, approaches, and resources. In this sense, biomimicry has shown itself to be an area of great potential for projects in design. In this work, the expression “biomimetic design” was generically adopted when analyzing publications from the intersection between the design and biomimicry domains. However, it is necessary to understand that further efforts are needed in favor of more ‘genuinely’ biomimetic designs, developed in the specific field of design, in which, throughout the entire creative process of products and services, the concepts, principles, and creative tools of biomimicry are effectively integrated.

Unlike technologies that require specific procedures, authorizations, investments, and monetization for access, nature is an extensive (and more accessible) repository of resources that can be investigated and interpreted by human beings, which can complement and expand current practices in design. Furthermore, as evidenced by several authors, the field of design is plural and interdisciplinary, as it seeks to enable creations for different areas of human endeavor [1,2,3,4,50,51,52,53,54,55]. Including the perspective of biomimicry in teaching and professional practice scenarios in design can expand creative horizons, allowing the creation of innovative solutions that are more efficient, economical, and better adapted to consumer needs and environmental diversity [6,55,56].

Finally, as the study of aspects of biomimicry in design can enhance the development of multiple skills (such as creative and conceptual generation, observation and research, study of materials, graphic expression, and three-dimensional conception and digital modeling), it is important to recognize that understanding nature and knowing how to explore its resources are indispensable requirements for contemporary professional training, since they can encourage creativity as well as the integration of ecological and sustainable factors in production cycles.

## Figures and Tables

**Table 1:** Classification of information collected in the Web of Science database, distributed quantitatively into categories. Source: the authors.

Categories	Data from Web of Science
Knowledge areas with the highest number of contributions	Engineering (*n* = 64) Science of Technology (*n* = 41) Environmental Sciences and Ecology (*n* = 31) Architecture (*n* = 24)
Countries with the highest number of publications	United States (*n* = 68) United Kingdom (*n* = 19) Turkey (*n* = 15)
Journals that published the most works	WIT Transactions on Ecology and the Environment (*n* = 9) Scientific Reports (*n* = 6) Architectural Science Review (*n* = 5) Procedia Social and Behavioral Sciences (*n* = 5)
Institutions that published the most works	University of California System (*n* = 8) Victoria University of Wellington (*n* = 8) University System of Georgia (*n* = 7) Delft University of Technology and University of Akron (*n* = 6)
Authors who published the most works	Maibritt Pedersen Zari (*n* = 8) Donnison, Faludi, Jones, Niewiarowski and Pauw (*n* = 3)
Years with the highest number of publications	2019 (*n* = 27) 2016 (*n* = 26) 2020 (*n* = 24)

**Table 2:** Synthesis of works with 35 or more citations registered in the Web of Science. Source: the authors.

Title	Citations	Main Contributions of the Author(s)
“Biomimetics: lessons from nature—an overview” [26]	691	Biomimetic approaches on a microscopic scale, inspired by morphological and physicochemical properties for the development of materials, devices, and surfaces were identified. The authors gathered a set of inspiring natural elements and pointed out applications in products.
“Biomimicry in textiles: past, present and potential —an overview” [27]	81	Characteristics of nature relevant to the field of bio-inspired textile design were highlighted: fiber diversity (strength, structure); functional surfaces (adhesion, hydrophobicity); thermal insulation and optical systems (structural colors and photonic materials).
“Microstructured barbs on the North American porcupine quill enable easy tissue penetration and difficult removal” [28]	69	Applications based on the defensive strategies of the species *Erethizon dorsatum* (pointed and sharp dorsal structures) were examined. The findings demonstrated good adherence and reduced the force required for penetration into tissues, which could be adopted in the design of hospital products (e.g., needles).
“Design and fabrication of multi-material structures for bioinspired robots” [29]	63	Multi-material rapid prototyping processes were studied for biomimetic robot design. Uses of stiff and flexible materials and the incorporation of sensors were discussed, in which nature-based configurations demanded less active control compared to traditional procedures.
“Integrating backcasting and eco-design for the circular economy the BECE framework” [30]	61	The authors underlined which aspects of the circular economy can mitigate the impacts of environmental degradation and presented the framework Backcasting and Eco-Design for the Circular Economy. Investigations on nature-based notions and areas were identified (biomimicry, cradle-to-cradle, natural capitalism, regenerative design).
“Evolution of reaction center mimics to systems capable of generating solar fuel” [31]	51	Artificial reactions based on photosynthesis to produce fuels through photochemical processes were mapped. Research on photoelectric systems capable of converting water into oxygen and hydrogen was evidenced.
“Templates and anchors for antenna-based wall following in cockroaches and robots” [32]	46	The authors highlighted that natural factors stimulate innovations in robot design and neuromechanics. An antenna project for task control (guiding the angulation of robots in relation to walls), based on the spatial navigation of the *Periplaneta americana* species, was illustrated.
“Biomimetic self-cleaning surfaces: synthesis, mechanism and applications” [33]	43	The study focused on the self-cleaning capacity of organisms by classifying them based on the use or the absence of water. It was found that new design projects inspired by these characteristics can be applied in: medicine, aerospace construction, solar energy production, and water treatment.
“Biomimetic design for climate change adaptation and mitigation” [34]	39	Systems-level regenerative and biomimetic design to mitigate the causes and effects of climate change was examined. The importance of renewable energies, responsive systems, local context adaptation, feedback loops, and autonomy was noted. Uses of biomimetic principles, in short, medium, and long-term projects were mentioned.
“A model based on Biomimicry to enhance ecologically sustainable design” [15]	35	The study measured and summarized biomimicry applications in environmental preservation projects in design and architecture. The following tools were explored: BioTRIZ, Typological Analysis, Nature Studies Analysis, and Biomimetic Spirals (Biomimicry Thinking Design Lens).

## Data Availability

Not applicable.
